# In-House Developed ELISA Indicates High Prevalence of Anti-*Echinococcus granulosus* IgG in Sheep Population—An Update from Pakistan

**DOI:** 10.3390/pathogens9110905

**Published:** 2020-10-29

**Authors:** Mughees Aizaz Alvi, John Asekhaen Ohiolei, Li Li, Muhammad Saqib, Muhammad Hammad Hussain, Muhammad Haleem Tayyab, Muzafar Ghafoor, Warda Qamar, Noman Yousaf Faridi, Anum Aizaz Alvi, Bao-Quan Fu, Hong-Bin Yan, Wan-Zhong Jia

**Affiliations:** 1 State Key Laboratory of Veterinary Etiological Biology, National Professional Laboratory of Animal Hydatidosis, Lanzhou Veterinary Research Institute, Chinese Academy of Agricultural Sciences, Lanzhou 730046, China; mugheesaizazalvi@gmail.com (M.A.A.); asekhaenj@gmail.com (J.A.O.); lili03@caas.cn (L.L.); fubaoquan@caas.cn (B.-Q.F.); yanhongbin@caas.cn (H.-B.Y.); 2 Department of Clinical Medicine and Surgery, University of Agriculture, Faisalabad 38400, Pakistan; drsaqibm@uaf.edu.pk (M.S.); drmhkhan381@gmail.com (M.H.T.); muzafar1512@gmail.com (M.G.); 3 Independent Researcher, Bardia, NSW 2565, Australia; m.hammad.hussain@gmail.com; 4 Department of Pathobiology, University of Veterinary and Animal Sciences, Lahore Sub-Campus Jhang 12-Km Chiniot Road, Jhang 35200, Pakistan; wardaqamar17@gmail.com; 5 Punjab Livestock and Dairy Development, Government of Punjab, Chiniot 35400, Pakistan; nomanfaridi786@gmail.com; 6 Independent Researcher, Faisalabad 38000, Pakistan; anumzgalaxy@gmail.com

**Keywords:** *Echinococcus granulosus*, ELISA, epidemiology, seroprevalence, sheep, Pakistan, risk factor

## Abstract

Cystic echinococcosis (CE) is a World Health Organization (WHO)-listed neglected tropical farm economy jeopardizing and public health concern disease. This study was aimed at furnishing sero-epidemiological baseline data of CE in sheep in Pakistan, where data are non-existent. For this purpose, two sheep-rich provinces of Pakistan were selected, and 728 sheep sera were collected using probability proportional to size (PPS) statistical technique. Epidemiological information was recorded on a questionnaire for the estimation of potential risk factors. The serum samples were analyzed for IgG antibodies against *Echinococcus granulosus* using an in-house-developed EgAgB-based ELISA kit. The overall seroprevalence recorded was 21.98% (160/728) in the tested sheep, suggesting higher seropositivity in sheep from Punjab (23.73%) as compared to Khyber Pakhtunkhwa (KPK) (19.04%). The overall apparent prevalence observed by this ELISA method was almost similar to the calculated true prevalence (21.77%). Prevalence was significantly different (*p* < 0.05) among sheep from different districts. Higher prevalence was found in females (22.54%, OR 1.41), age group > 5 years (29.66%, OR 1.64), crossbreeds (42.85%, OR 2.70), and sheep with pasture access (25.96%, OR 3.06). Being in age group > 5 years and having pasture access were the factors significantly associated with seropositivity (*p* < 0.05). This study provides serological evidence of *E*. *granulosus* infection in sheep and can be used as a model for *ante-mortem* screening of the sheep globally.

## 1. Introduction

Cystic echinococcosis (CE) is a zoonotic parasitic disease caused by the metacestodes of *Echinococcus* belonging to the family *Taeniidae.* The metacestode is made up of a cystic structure consisting of, from inside to outside, the hydatid fluid, the germinal layer producing the protoscoleces, parasitic laminated layer, and the adventitial layer, fabricated as a result of the host’s immune response [1]. They infect a wide spectrum of animal species including livestock and wildlife [2,3,4,5]. The cosmopolitan distribution of CE has led to losses of three billion USD annually [6]. *Echinococcus granulosus* (*sensu lato*) complex contains at least ten valid strains/genotypes. Of them, *E*. *granulosus sensu stricto* (G1–G3) is the most important as it is responsible for the majority of the global CE burden. The definitive hosts of this cestode are the canids that carry the adult tapeworm parasite in their small intestines. Both wild and domesticated ruminants, including sheep, serve as intermediate hosts [7]. Fertilized eggs are released from the intestine of dogs in feces and ingestion of contaminated water or vegetation by a suitable intermediate host leads to the release of oncospheres from embryonated eggs that penetrate the intestinal wall, spreading to various tissues of the body through the circulatory system [8,9,10].

The course of the disease is mostly asymptomatic until there is large cyst formation. The formation of hydatid cysts primarily occurs in the liver and lungs, and ingestion of such infected carcasses by dogs leads to completion of the life cycle [11,12,13]. The World Health Organization (WHO) classifies CE cysts into three types: active cysts (stage CE1 and CE2), transitional cysts (stage CE3), and degenerating or inactive cysts (stage CE4 and stage CE5) [14]. The treatments regimens that are adopted depending upon the stage of the cyst include surgical resection of the cystic mass, percutaneous drainage with protoscolecides, and the anthelmentic drugs like albendazole, while the response to the treatment is highly variable [15].

Cystic echinococcosis encompasses a wide geographical area from Eastern parts of Asia to Northern America and from the upper northern hemisphere to southern countries of the African continent [16,17]. This malady is included in the WHO list of neglected tropical diseases of public health concern. Eurasia, Australia, Africa and South America have a very high prevalence of the disease, and an estimated 50 million people are infected with CE worldwide [18]. This disease plays havoc with the economy of the livestock industry in endemic countries in terms of treatment cost, production losses and, in some cases, mortality of infected animals and aberrant human host infection are the economic and social thrashes resulting from this infection. It has been estimated that CE causes losses of up to USD 276.20 per 100 infected goats and sheep and USD 165.72 per 100 infected large ruminants and camels. Losses occur in terms of reduced quantity and quality of milk, wool, and meat production as a result of retarded growth, drop in fertility rate and finally condemnation of infected carcasses [19].

Pakistan is an agricultural country where the livestock industry contributes 11.4% to the overall Gross Domestic Product (GDP) of the country. There are at least 30.1 million heads of sheep according to the recent animal census [20]. To the best of our knowledge, to date, no comprehensive sero-epidemiological study on ovine CE has been conducted, despite the fact that this disease has been reported from neighboring countries like China, India and Iran. Keeping in view the high population of sheep in the country and the magnitude of economic losses worldwide, this study was designed to assess the seroprevalence and associated risk factors of sheep CE in two provinces of Pakistan.

## 2. Results

**Figure 1:**
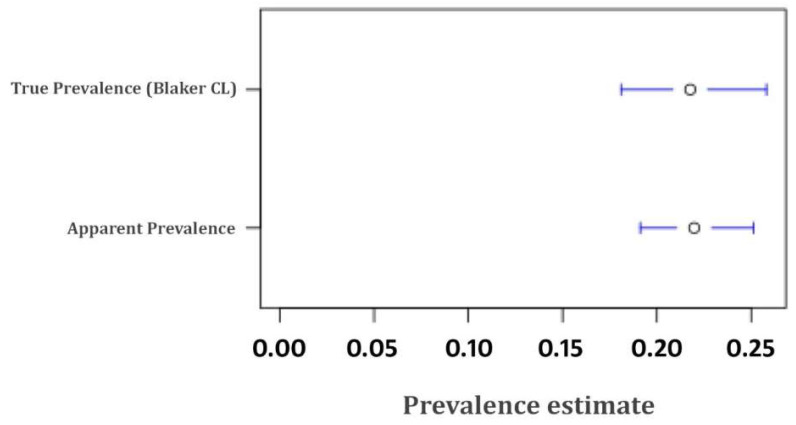
The difference between percentage and Balker confidence interval observed for apparent prevalence and true prevalence.

Overall, 108 (23.73%; 95% CI = 19.9–27.9) and 52 (19.04%; 95% CI = 14.6–24.2) sheep were found positive for anti-*Echinococcus granulosus* IgG from Punjab and KPK Province, respectively. The seroprevalence of *E*. *granulosus* (23.73%; 95% CI = 19.9–27.9) in sheep was higher in Punjab province, however, the difference recorded between the provinces was not significant (*p* > 0.05). The difference between percentage and Balker confidence interval observed for apparent prevalence and true prevalence estimated is shown in Figure 1.

**Figure 2:**
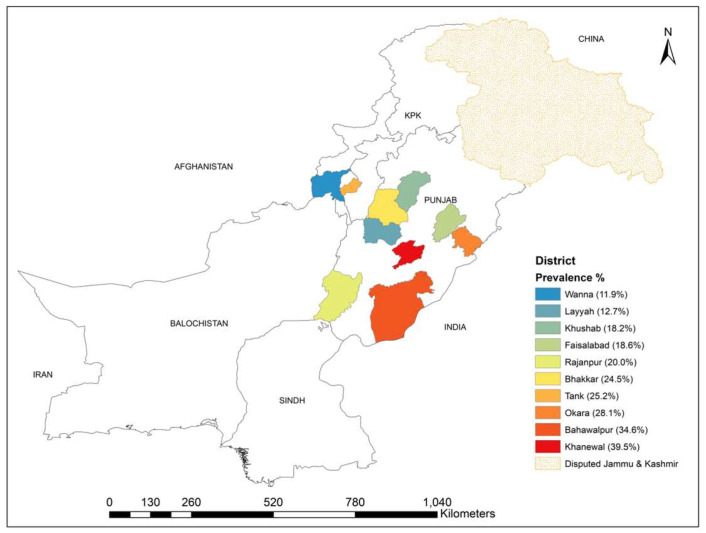
Prevalence percentage of anti-*Echinococcus granulosus* IgG in study areas.

District-wise prevalence results are given in Table 1 and Figure 2. From Punjab, the highest prevalence was recorded at Khanewal district (39.47%; 95% CI = 24–56.6%), while the lowest prevalence was observed in sheep hosted at Layyah district (12.72%; 95% CI = 5.3–24.5%). From KPK province, the prevalence in Tank and Wana agencies were 25.17% and 11.90%, respectively.

The influence of individual-level variables (age, sex, breed, and pasture access) on the seroprevalence of *E*. *granulosus* in sheep is summarized in Table 2. The analysis of individual-level variables indicated that older sheep (>5 year) (OR: 1.64; 95% CI = 1.05–2.54; *p* = 0.029) were more likely to test positive than young sheep (≤5 year). Female sheep (22.54%; 95% = CI 19.4–26; OR 1.41) and crossbreeds (42.85%; 95% CI = 9.9–81.6%; OR 2.70) were more seropositive, however, no significant statistical difference (*p* > 0.05) was observed. Sheep with pasture access showed more seropositivity (25.96%; 95% CI= 22.3–29.9; OR 3.06) and a statistically significant difference (*p* < 0.05) was observed between grazing and lot-fed sheep.

All variables with *p-*value < 0.020 in the univariable analysis were used to construct a binary logistic regression model to predict the seropositivity in sheep. A backward stepwise approach was used and variables with *p-*value > 0.05 were removed from the model at subsequent steps until all significant variables remained in the final model. The province, breed and sex were removed at subsequent steps while grazing/pasture access remained in the final model. Therefore, no multivariable model was left to fit our data.

## 3. Discussion

In the current study, EgAgB fraction-based, in-house indirect ELISA was developed to determine the prevalence of anti-*Echinococcus granulosus* IgG in sera of sheep hosted in two provinces of Pakistan. Prior to this study, no report on the seroprevalence of *E. granulosus* in Pakistani sheep was available. Thus, this study provides a broad outlook on CE prevalence in sheep population reared in Pakistan.

The overall seroprevalence in two provinces of Pakistan (Punjab 23.73%; KPK 19.04%) was found to be 21.98%. True prevalence [21] and 95% CI Blaker’s method [22] (21.77%; 95% CI 18.10–25.81) were very similar to the apparent prevalence (21.98%) observed based on an in-house indirect ELISA.

Non-significant association (*p* > 0.05%) was found between seroprevalence and location, which is in line with the findings of Pour et al. [23], who observed a statistically non-significant difference (*p* > 0.05) in prevalence between Khuzestan (9.9%) and Ardabil provinces (8%) of Iran. However, a significant difference (*p* < 0.05) in the prevalence of CE between sheep from different study districts was observed, which is concomitant with the remarks of Qingling et al. [24], who reported the highest infection rate at Yining slaughterhouse (12.5%), and the lowest at Urumqi slaughterhouse, with significant differences (*p* < 0.05).

The results also demonstrate a higher percentage of seropositivity in female animals (22.54%) compared to their male counterparts (17.10%), and the odds of testing seropositive were also higher in females (OR 1.41). However, this observation could be biased for several reasons. Firstly, a higher fraction of the sera were collected from female sheep as compared to male sera samples. Secondly, female sheep generally enjoy a longer life span, as they are reared for breeding and milk purposes while males are slaughtered at a younger age. Some surveys show that there is no association between the prevalence of CE and the sex [25], while many studies have demonstrated a higher prevalence in female animals compared to males [26,27,28].

In this study, increasing age was also observed as one of the most important risk factors for CE among sheep in Pakistan. A positive correlation between prevalence and age was observed. Higher seropositivity was found in sheep >5 years of age compared to younger age groups. The odds of testing positive were found higher in older animals (OR 1.64). This conforms to the observations of Ibrahim [28] and Fathi et al. [29]. Age as a potential risk factor for CE in the current study is in accordance with the results of Li et al. [30], Pour et al. [23], Islam [31], Mitrea et al. [32], and Elham et al. [8] who reported that age positively correlated with infection. The justification of a positive correlation of age may be adopted from the findings of Torgerson et al. [33], who developed a model to describe the relationship and association between age and number of protoscolices. The model revealed that as sheep age, there is an increase in the number of protoscolices. It was reported that an infected 4-year-old sheep has abundantly higher (up to 9700) protoscolices than younger sheep (16 protoscolices) [33]. This situation thus increases the likelihood of being seropositive.

To the best of our knowledge, breed susceptibility has not been reported in the case of natural infections. However, experimental infection of Angora goats in Australia found this breed to be more susceptible in contrast to undomesticated goats [34]. The results of this study showed that cross-bred goats were more sero-positive than local/indigenous breeds.

Higher prevalence of CE in grazing sheep in the current study may have resulted due to exposure of sheep to dog fecal material carrying *E*. *granulosus* eggs that otherwise contaminate grasses and water. Evidence of pasture contamination as one of the major factors influencing the distribution and prevalence of *E. granulosus* has also been documented [26].

EgAgB is a potential candidate for the development of ELISA for estimating the exposure of herds to *E*. *granulosus* [35,36,37,38]. The specificity of the test developed was high and in accordance with previously developed EgAgB-based ELISA of sheep origin [26]. A small difference (0.21%) was found between the observed apparent prevalence and true prevalence [21].

## 4. Materials and Methods

### Study Locales and Sample Collection

**Figure 3:**
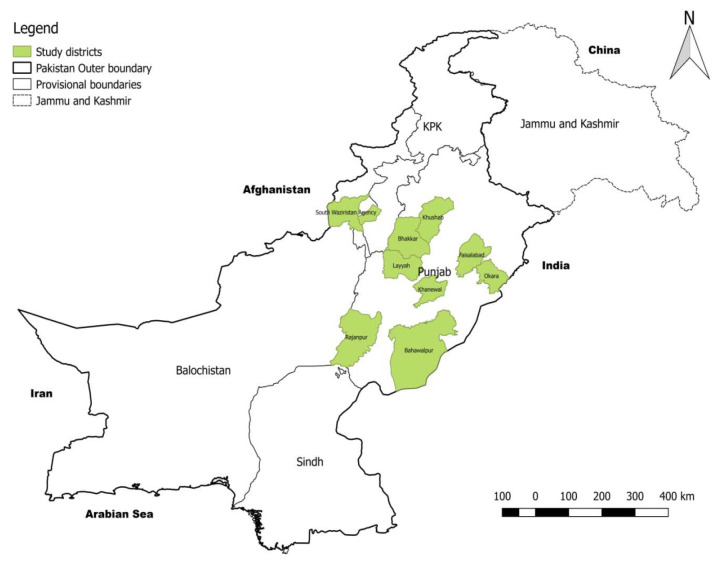
Map showing sheep sera sampling sites in Punjab and KPK provinces of Pakistan.

The study encompassed broad geographical areas covering eight districts of Punjab and two agencies of the Khyber Pakhtunkhwa (KPK) province (Figure 3). From Punjab, blood was collected from 652 sheep hosted at different Livestock Experimental Stations (LES) situated in different districts by proportionate sampling method (Table 3), and 150 samples were collected from sheep raised in district Faisalabad through convenient random sampling. From KPK province, two agencies of South Waziristan, namely Wana and Tank, were included in this study because of the high population of sheep and proximity to Afghanistan, from where human CE reports are available. A total of 600 samples (300 from each agency) were collected through random sampling.

Blood from the juglar vein was collected aseptically using 5 mL sterile syringe following proper restraining protocol, and blood was added in a gel-clot activator vacutainers to separate serum (Improvacuter, Hamburg, Germany) and labeled accordingly. All the samples were brought to the laboratory and centrifuged at 4000 rpm for 10 min to obtain the maximum amount of sera. Sera were transferred to cryovials and stored at −20 °C until further use [39]. Information regarding animal data and variables were also recorded during sample collection on the pre-designed questionnaire.

## 5. ELISA Development

### 5.1. Positive and Negative Control Sera

Validation of in-house-developed ELISA was made by testing the 200 positive and negative sera. Positive sera (*n* = 200) were collected from sheep harboring *E*. *granulosus* cyst, either in their liver or lungs, while the molecular identity of *E*. *granulosus* was confirmed through the amplification of a segment of the mitochondrial *cox*1 gene [40] and subsequent sequencing analysis. Similarly, negative sera were collected from experimental sheep raised in confinement and had not demonstrated any lesions related to CE upon necropsy. For cross-reactivity of in-house ELISA with other cestodal diseases, sera from 20 *Cysticercus cerebralis* (*Taenia multiceps*) and 20 *C. tenuicollis* (*T*. *hydatigena*) positive sheep were included in the testing.

### 5.2. Sheep Hydtaid Cystic Fluid

Sheep hydatid cystic fluid (HCF) was withdrawn aseptically from pulmonary and hepatic cysts of recently slaughtered sheep. All the sheep were naturally infected with *E*. *granulosus* and confirmation was done through PCR. The HCF was subjected to centrifugation at 2000× *g* for 20 min at 4 °C to sediment the protoscolices. A clear supernatant HCF was collected in sterilized falcon tubes and stored at −70 °C till further use.

### 5.3. EgAgB Preparation

The clear HCF was used to prepare the EgAgB-enriched fraction, as described previously by Ibrahem et al. [41] and Oriol et al. [42]. Briefly, overnight dialysis of 100 mL clarified HCF was carried out overnight at 4 °C using 0.005 M acetate buffer (pH 5) followed by centrifugation at 15,000× *g* for 45 min at 4 °C. A 0.2 M phosphate buffer (10 mL; pH 8) was used to dissolve the pellet. The obtained suspension was boiled in a water bath for 15 min, followed by centrifugation at 20,000× *g* for 60 min at 4 °C. The supernatant contained EgAgB and the protein concentration was measured using the Biuret method. This EgAgB rich fraction was stored at −20 °C until use.

### 5.4. Optimization of EgAgB Indirect ELISA Test

The ELISA was optimised by performing checkerboard titrations, as described previously by Voller et al. [43]. The optimal working concentrations for EgAgB fraction and conjugate in combination with serially diluted positive and negative control sera were determined. Positive and negative control sera were obtained from animals that were infected with *E*. *granulosus* and animals raised under helminth free control conditions, respectively.

The cut-off value was calculated as absorbance values of Negative Control sera + 3× standard deviation. All tests were validated, as (i) the absorbance value of substrate blank was lower than 0.200 and (ii) the ratio of the mean values of positive controls and negative controls was greater than 2. Samples with OD ratio of sample/mean of negative controls ≥2 were considered positive, while samples <2 were considered negative.

The sensitivity and specificity of the *E*. *granulosus* detection kit was evaluated using 200 *E*. *granulosus* positive and 200 negative sheep sera and 20 *Cysticercus cerebralis* positive and 20 *C. tenuicollis* positive sheep sera and were 83% and 95%, respectively.

## 6. EgAgB iELISA Procedure

Out of the 802 sera from Punjab and 600 from KPK, a total of 455 and 273 samples, respectively, were selected by randomization without replacement using an online available epidemiological tool [44]. The 96-well microtitration plates were coated with 100 μL purified EgAgB (0.5µg protein per well) diluted in 0.05 M carbonate/bicarbonate buffer (pH 9.6). Overnight incubation was carried out at 4 °C, followed by three times washing of the plates with 0.1% PBS and 0.05% Tween-20 (PBS-T, pH 7.4). After washing, each well was blocked with 300 µL of 0.3% PBS-T (contains 1% casein) and incubated at 37 °C for 1 h. Afterwards, the blocking solution was discarded and 100 µL of diluted (1/100 in 0.3% PBS-T) positive, negative and animal test sera were dispensed into the designated wells and incubated at room temperature for 1 h. This step was followed by washing as described previously and 100 µL of peroxidase-conjugated Protein G (LSI VET, Lissieu, France) (at dilution of 1:10,000) was added into each well and incubated at room temperature for 45 min. The plate was washed again and 100 µL TMB substrate was dispensed into each well. The reaction was terminated by the addition of 50 μL of 2 M sulphuric acid after 15 min and absorbance values were recorded using a microplate reader at 450 nm.

Data collected were categorized and prevalence was calculated at a 95% confidence interval (CI) [45]. Moreover, the estimate of true prevalence was calculated as described previously [21]. Chi-square (χ2) test was performed to calculate the significance of association (*p* < 0.05) between different variables. Bivariable analysis was conducted and odds ratio (OR) along with 95% CI was calculated for each variable. Finally, a multivariate logistic regression analysis was conducted to assess the association between seroprevalence and variables found to be significant (*p* < 0.20) in the initial bivariable screening. Data were analyzed using IBM SPSS Statistics 17.0 for Windows^®^ (IBM Corporation, Route 100 Somers, New York, NY, USA).

## 7. Conclusions

This sero-survey conducted for the very first time in Pakistan reveals the endemicity and high prevalence of cystic echinococcosis among sheep population in the country. The seroprevalence is higher compared to reports from other countries with age and grazing patterns observed as potential risk factors. Sero-assays like ELISA can be a valuable tool for herd-level screening and surveillance to design effective control programs. Reinforcing zoonotic disease close-watch, including the collection of field samples for molecular characterization of prevalent genotypes, is highly warranted in future perspectives.

## Figures and Tables

**Table 1:** Prevalence of *E*. *granulosus* in the sheep sampled from different farms in Punjab and Khyber Pakhtunkhwa (KPK) provinces of Pakistan.

Province	District	Positive/Tested	Prevalence% (95% CI)
Punjab	Khanewal	15/38	39.47 (24–56.6)
	Bhawalpur	9/26	34.61 (17.2–55.7)
	Okara	18/64	28.12 (17.6–40.8)
	Bhakkar	35/143	24.47 (17.7–32.4)
	Rajanpur	2/10	20.00 (2.5–55.6)
	Faisalabad	16/86	18.60 (11–28.4)
	Khushab	6/33	18.18 (7–35.5)
	Layyah	7/55	12.72 (5.3–24.5)
KPK	Tank	37/147	25.17 (18.4–33)
	Wana	15/126	11.90 (6.8–18.9)
Total		160/728	21.98 (19–25.2)

**Table 2:** Univariable analysis of different risk factors for the seroprevalence of *E. granulosus* in sheep sampled from Punjab and KPK provinces of Pakistan.

Variable	Category	Pos./Tested	Prev.% (95% CI)	Significance	OR	95% CI	*p* Value
Province	Punjab	108/455	23.73 (19.9–27.9)	χ^2^ = 2.187 *p* = 0.139	1.32	0.91–1.92	0.14
	KPK	52/273	19.04 (14.6–24.2)		Ref	-	
Age group	>5 Year	35/118	29.66 (21.6–38.8)	χ^2^ = 4.848 *p* = 0.028	1.64	1.05–2.54	0.029
	≤5 Year	125/610	20.49 (17.4–23.9)		Ref	-	
Sex	Female	147/652	22.54 (19.4–26)	χ^2^ = 1.175 *p* = 0.278	1.41	0.76–2.64	0.28
	Male	13/76	17.10 (9.4–27.5)		Ref	-	
Breed	Cross	3/7	42.85 (9.9–81.6)	χ^2^ = 1.796 *p* = 0.183	2.70	0.60–12.16	0.197
	Local	157/721	21.77 (18.8–25)		Ref	-	
Pasture access	Yes	141/543	25.96 (22.3–29.9)	χ^2^ = 19.826 *p* < 0.001	3.06	1.84–5.11	<0.001
	No	19/185	10.27 (6.3–15.6)		Ref	-	

**Table 3:** Population and proportionate sampling from Livestock Experimental Stations, Punjab, Pakistan.

Sr No.	Experimental Station/Farm	At Farm Sheep Population	Percent of Total Sheep Population	Samples Collected
1	LES AlladadJahnia	436	10.5	46
2	LES Khushab	650	15.7	102
3	LES Fazilpur Farm	72	1.7	1
4	LES Jogaitpur	400	9.7	39
5	LES RakGhulama	93	2.2	2
6	LES RakKharewala	757	18.3	138
7	LES Bahadarnagar	1050	25.4	267
8	GLF Kallorkot	380	9.3	35
9	Fine Wool Sheep Farm	300	7.2	22
Total		4138	100.00	652

LES = Livestock Experimental Station; GLF = Government Livestock Farm.
